# Comparative Analysis of the Quality of Life Among Men With Premature Ejaculation and Erectile Dysfunction at a Tertiary Care Hospital in Bangladesh

**DOI:** 10.7759/cureus.83849

**Published:** 2025-05-10

**Authors:** Mohammad Shamsul Ahsan, Leuza Mubassara, Md. Mahbubul Hasan, Jebun Nahar, Rubaiya Ali, Ananya Kar, Fatema Tuj Johora, Moumita Paul

**Affiliations:** 1 Department of Psychiatry, Bangabandhu Sheikh Mujib Medical University, Dhaka, BGD; 2 Department of Psychiatry, Jashore Medical College Hospital, Jashore, BGD; 3 Department of Psychiatry, National Institute of Mental Health, Dhaka, BGD; 4 Department of Dermatology and Venereology, Evercare Hospital, Dhaka, BGD

**Keywords:** depression, erectile dysfunction, mental health, physical health, premature ejaculation, quality of life, sexual health

## Abstract

Background: Premature ejaculation (PE) and erectile dysfunction (ED) are among the most prevalent sexual disorders in men, significantly impairing quality of life (QoL). This study aimed to assess and compare QoL among patients with PE and ED.

Methods: A cross-sectional, single-center study was conducted where participants aged ≥18 years with complaints of either PE or ED were included, while those with both conditions were excluded. PE, ED, and QoL, along with depression, were assessed using validated tools. All statistical analyses were conducted using IBM SPSS Statistics software, version 26 (IBM Corp., Armonk, NY). A two-tailed p-value of <0.05 was considered statistically significant. Continuous variables (e.g., QoL domain scores) were summarized using means and standard deviations (SD). Differences in QoL domain scores between patients with PE and ED were analyzed using one-way ANOVA. An independent samples t-test was used for the unadjusted analysis to compare mean differences in World Health Organization Quality-of-Life Scale Brief Version (WHOQOL-BREF) domain scores between the PE and ED groups. For the adjusted analysis, analysis of covariance (ANCOVA) was employed, controlling for potential confounders including depression severity (Patient Health Questionnaire-9 (PHQ-9)), physical illness, smoking status, physical activity, and education level.

Results: Among the 245 participants, 140 (57.1%) presented with PE and 105 (42.9%) with ED. Based on diagnostic tools, 87.1% of PE cases were confirmed using the Bengali-validated Premature Ejaculation Diagnostic Tool (PEDT), and 95.2% of ED cases were confirmed using the IIEF-6. Both groups scored highest in the physical domain and lowest in the social domain of WHOQOL-BREF, with PE generally showing better scores. Depression was prevalent in both groups, affecting 72.9% of PE and 65% of ED participants, with severe depression slightly more common in ED (7%). Physical illnesses like hypertension and diabetes were more frequent in ED patients. Education level, active lifestyle, smoking status, and living place (urban vs. rural) were significantly linked to the environmental and psychological domains (p = 0.028, p = 0.008, p = 0.013, p = 0.030). A history of physical illness significantly impacted the physical domain (p = 0.032). Statistical analyses revealed that PE participants had significantly better physical and psychological QoL scores than ED participants, both before and after adjusting for confounders such as depression severity (PHQ-9), physical illness, smoking status, physical activity, and education level.

Conclusion: This study highlights that men with PE report significantly better physical and psychological QoL compared to those with ED, even after adjusting for key confounding factors. Depression and physical illnesses were more prevalent among ED patients, negatively influencing their QoL. Sociodemographic and lifestyle variables also played a notable role in shaping QoL outcomes. These findings underscore the need for targeted interventions addressing both sexual dysfunction and associated psychosocial factors.

## Introduction

Sexual health represents a critical determinant of overall quality of life (QoL), rather than merely a lifestyle concern. Among male sexual dysfunctions, premature ejaculation (PE) and erectile dysfunction (ED) emerge as the most prevalent conditions. Epidemiological data indicate PE affects 20% to 40% of men globally, characterized by three core features: unintentionally rapid ejaculation, impaired ejaculatory control, and resultant distress for both partners [1]. ED, defined as the consistent inability to achieve or maintain an adequate erection for sexual activity, demonstrates an age-related progression, affecting approximately 30% of younger men and escalating to 52% among those aged between 40 and 70 years [2]. Men are particularly concerned as it affects their partner’s satisfaction, which in turn impairs their QoL, self-confidence, and self-respect. They often struggle to establish new relationships. The World Health Organization (WHO) conceptualizes QoL as an individual's comprehensive self-assessment of their physical, psychological, social, and environmental functioning within the context of health status [3]. This multidimensional construct encompasses four primary domains: physical well-being, mental health status, interpersonal relationships, and environmental adaptation capacity. Both PE and ED have been shown to substantially compromise these QoL dimensions, with particular emphasis on psychological distress and relationship quality.

PE has significant negative impacts on QoL; previous studies have shown that individuals experiencing premature ejaculation exhibited a lack of bodily acceptance and expressed dissatisfaction with their sexual experiences, with 49% of subjects reporting it as a problem and 65% expressing sexual dissatisfaction [4, 5]. PE can lead to frustration, embarrassment, dissatisfaction, feelings of incompetence, and depression. Studies have also revealed that men suffering from PE tend to have lower levels of intimacy between couples compared to those without PE, thereby hampering the social, emotional, and intellectual aspects of their lives [6]. Subjects with PE, compared to those without, had noticeably lower mean scores for perceived control over personal distress and difficulties related to ejaculation [6, 7]. Men with ED, particularly younger men, develop poorer QoL, especially when comorbidities are present, and they often avoid entering new relationships [8]. PE and ED are common but underreported sexual disorders, especially in conservative, religious countries like Bangladesh, where cultural stigma limits open discussion. These conditions negatively impact not only sexual satisfaction but also overall physical, psychological, and social well-being. Due to societal taboos and a lack of awareness, men often avoid seeking help, resulting in inadequate treatment [9]. While global studies have explored the QoL impacts of PE and ED, comparative data from the Bangladeshi context remain scarce. This study addresses that gap by evaluating and comparing QoL in men with PE and ED and examining the influence of depression and sociodemographic factors.

## Materials and methods

Study design and participants

This cross-sectional study was conducted in the Department of Psychiatry at Bangabandhu Sheikh Mujib Medical University (BSMMU) in Dhaka, Bangladesh, from January 2022 to October 2022. Men aged ≥18 years, sexually active in the previous month, who came with a complaint of PE or ED were included using a convenience sampling technique. Patients with serious mental illness, neurological disorders, or inability to provide a proper history were excluded.

Sample size

The study included 245 participants (approximately 122 per group), which provided sufficient power (80%) at a 5% significance level to detect a small-to-moderate effect size (Cohen’s d ≈ 0.38) in QoL scores between patients with PE and ED, based on standard sample size estimation formulas for two independent groups.

Data collection tool

A semi-structured questionnaire was employed to collect data on sociodemographic and related variables. PE was assessed using the Bengali-validated Premature Ejaculation Diagnostic Tool (PEDT), a standardized instrument comprising five items that assess aspects such as ejaculatory control, frequency, response to minimal stimulation, associated distress, and interpersonal difficulties. The total score ranges from two to 22, with scores above 11 indicative of PE, scores between nine and 11 suggesting probable PE, and scores below nine indicating no PE. The Bangla version demonstrated good psychometric properties, with a Cronbach’s alpha of 0.827 indicating high internal consistency. Test-retest reliability was strong, with a Spearman’s rho of 0.87 for total score and an intraclass correlation coefficient (ICC) of 0.943. Construct validity was confirmed via exploratory factor analysis, with a Kaiser-Meyer-Olkin (KMO) value of 0.77 supporting sampling adequacy [10].

ED was assessed using the Bengali version of the International Index of Erectile Function-6 (IIEF-6), where scores ≤25 signify varying degrees of ED. Factor analysis identified five underlying domains: erectile function, orgasmic function, sexual desire, intercourse satisfaction, and overall satisfaction, all with eigenvalues >1.0. The instrument showed high internal consistency, with domain-specific Cronbach’s alpha values ranging from 0.73 to 0.91. Test-retest reliability for each domain was also statistically significant [11]. Depressive symptoms were measured using the Patient Health Questionnaire-9 (PHQ-9), a validated nine-item Likert scale. A cutoff score of ≥10 showed both sensitivity and specificity of 88% for detecting major depression, with score thresholds of five, 10, 15, and 20 indicating mild, moderate, moderately severe, and severe depression, respectively [12]. QoL was evaluated using the Bengali-validated World Health Organization Quality-of-Life Scale Brief Version (WHOQOL-BREF), a 26-item instrument assessing four domains, namely, physical health, psychological well-being, social relationships, and environmental context, along with two general items on overall QoL and health. Higher scores denote better QoL. The scale showed satisfactory internal consistency: Cronbach’s alpha was 0.84 (physical), 0.84 (psychological), 0.62 (social), 0.66 (environmental), and 0.91 for the total score [13, 14].

Procedure of data collection

The study approval was obtained from the BSMMU Institutional Review Board (date: December 1, 2021; number: BSMMU/2021/13013). Participants were informed about the purpose, method, and outcome of the study. Inclusion criteria were clearly defined to enroll adult male patients (≥18 years) presenting with either PE or ED. Patients with both conditions were excluded to avoid misclassification and selection bias, and validated and standardized tools were used to avoid measurement bias.

Written informed consent was obtained from all participants after clearly explaining the purpose of the study, ensuring confidentiality, and emphasizing voluntary participation. No financial or material incentives were offered to the respondents. Participation was entirely voluntary, and respondents were assured that they could withdraw at any time without any consequences. To encourage honest disclosure in light of the cultural sensitivity around sexual health topics in Bangladesh, interviews were conducted in a private setting by trained clinicians of the same gender, with strict assurance of anonymity and non-judgmental communication.

PEDT was applied to all participants who complained of PE; those who scored >11 were diagnosed with PE, and sociodemographic data were collected from them by using the semi-structured questionnaire to identify the characteris­tics of the participants. IIEF-6 was applied to all participants who complained of ED; those who scored ≤25 were diagnosed with ED, and sociodemographic data were collected from them by using the semi-structured questionnaire to identify the characteris­tics of the participants. Then, the Bengali versions of PHQ-9 and WHOQOL-BREF were applied to diagnose and evaluate the severity of depression and to assess the QoL among patients, respectively. Data were collected by face-to-face interviews. The researcher read out the questions and marked the answers given by the participants who were not able to read. The participants had the right to stop the interview at any time, even without having to give a reason for stopping it. All participant data were de-identified by assigning unique codes instead of using personal identifiers (e.g., names, contact details), ensuring anonymity.

Data analysis

Data were analyzed by IBM SPSS Statistics software, version 26 (IBM Corp., Armonk, NY). Frequencies (n) and percentages (%) were used to show sociodemographic characteristics of participants, including the severity of depression, presence of physical illness, and severity of PE and ED. Descriptive statistics (mean ± SD and min-max) were used for QoL scores in dif­ferent domains. Differences in QoL domain scores between patients with PE and ED were analyzed using one-way ANOVA. An independent samples t-test was used for the unadjusted analysis to compare mean differences in WHOQOL-BREF domain scores between the PE and ED groups. For the adjusted analysis, analysis of covariance (ANCOVA) was employed, controlling for potential confounders including depression severity (PHQ-9), physical illness, smoking status, physical activity, and education level.

## Results

The classification of participants according to the severity of PE and ED, as assessed by validated screening tools, is presented in Table 1. Table 1 shows the distribution of participants according to their classification on the PEDT and IIEF-6 severity. Among 245 participants, 140 (57.1%) came with a complaint of PE and 105 (42.9%) with ED. Of the 140 patients, 122 (87.1%) were diagnosed with PE by the PEDT, and of the 105 patients, 100 (95.2%) were diagnosed with ED by the IIEF-6.

**Table 1:** Proportion of participants having PE and ED (N=245) PE: premature ejaculation; ED: erectile dysfunction; PEDT: Premature Ejaculation Diagnostic Tool; IIEF-6: International Index of Erectile Function – 6-item version

PEDT class	n (%)	IIEF-6 class	n (%)
No PE	6 (4.3)	No ED	5 (4.8)
Probable PE	12 (8.6)	Mild ED	22 (21)
PE	122 (87.1)	Mild to moderate ED	41 (39)
		Moderate ED	20 (19)
		Severe ED	17 (16.2)
Total	140 (100)		105 (100)

Table 2 shows the QoL score across different domains of WHOQOL-BREF among participants. The assessments revealed that both PE and ED participants scored highest in the physical domain (PE: 14.09; ED: 13.39) and lowest in the social domain (PE: 11.36; ED: 11.11).

**Table 2:** Quality of life of the participants across different domains of WHOQOL-BREF WHOQOL-BREF: World Health Organization Quality of Life Brief Version; PE: premature ejaculation; ED: erectile dysfunction

Domain	PE (n=122)		ED (n=100)	
	Mean±SD	Min-Max	Mean±SD	Min-Max
Physical	14.09 ±2.6	7-20	13.39 ±2.4	7-20
Psychological	12.42 ±2.7	4-19	11.67 ±2.7	7-19
Social	11.36 ±2.2	5-17	11.11 ±2.3	4-16
Environmental	13.17 ±2.2	7-20	13.11 ±2.1	9-20

**Figure 1:**
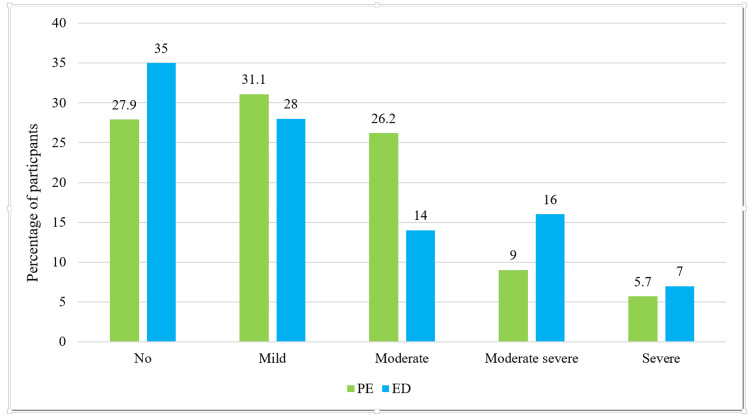
Percentage of participants showing different severity of depression on the PHQ-9 scale (n=222) PHQ-9: Patient Health Questionnaire-9; PE: premature ejaculation; ED: erectile dysfunction

Figure 1 shows the different levels of depression observed in diagnosed PE and ED patients. Of the PE patients, 72.9% showed different levels of depression, and of the ED patients, 65% showed different levels of depression. Depression severity varied, with 5.7% of PE and 7% of ED participants reporting severe depression.

**Figure 2:**
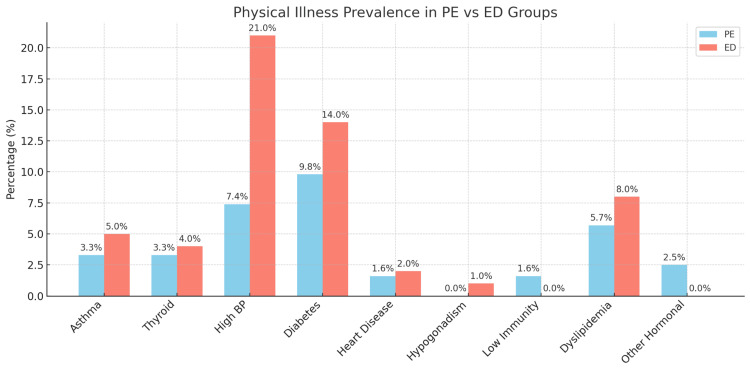
Percentage of participants having different physical illnesses (n=222) PE: premature ejaculation; ED: erectile dysfunction

Figure 2 shows participants with PE and ED having different physical illnesses. Hypertension was more prevalent among ED patients (21%) compared to PE patients (7.4%). Similarly, diabetes was observed in 14% of ED patients and 9.8% of PE patients.

Table 3 shows that several sociodemographic and clinical variables significantly affect QoL domains in participants with PE and ED. Depression severity was consistently associated with poor QoL across all domains for both conditions (PE: p < 0.0001, ED: p < 0.001). Education level, active lifestyle, smoking status, and living place (urban vs. rural) were significantly linked to the environmental and psychological domains (p = 0.028, p = 0.008, p = 0.013, p = 0.030). A history of physical illness significantly impacted the physical domain of QoL (p = 0.032).

**Table 3:** Sociodemographic and clinical variables with statistically significant effects on QoL domains in participants with PE and ED * p value < 0.05; ** p value < 0.01; ***p value < 0.001; only statistically significant associations (p < 0.05) from one-way ANOVA are shown. QoL: quality of life; PE: premature ejaculation; ED: erectile dysfunction

Domain of QoL affected	Associated variable(s)	PE p-value(s)	ED p-value(s)
Environmental	Education level	p = 0.028*	p = 0.028*
	Active lifestyle	p = 0.028*	p = 0.028*
	Smoking status	p = 0.008*	p = 0.008*
	Living place	p = 0.013*	p = 0.013*
	Depression severity	p = 0.001***	p = 0.001**
Psychological	Living place	p = 0.030*	p = 0.030*
	Depression severity	p < 0.0001***	p < 0.001***
Physical	Past history of physical illness	p = 0.032*	p = 0.032*
	Depression severity	p = 0.011*	p = 0.011*
Social	Depression severity	p < 0.001***	p < 0.001***

Table 4 shows that the unadjusted analysis revealed significant differences between the PE and ED groups in the physical (p = 0.030) and psychological (p = 0.032) QoL domains, with PE showing higher scores. After adjusting for depression severity, physical illness, smoking status, physical activity, and education level, these differences remained significant for the physical (p = 0.045) and psychological (p = 0.041) domains. However, no significant differences were found in the social (p = 0.392; p = 0.578) or environmental (p = 0.829; p = 0.765) domains, both unadjusted and adjusted.

**Table 4:** Unadjusted and adjusted mean differences in World Health Organization Quality of Life Brief Version (WHOQOL-BREF) domain scores between PE and ED groups * p value < 0.05; independent sample t-test used for unadjusted analysis; analysis of covariance (ANCOVA) used for adjusted analysis; **Adjusted for depression severity (Patient Health Questionnaire-9 (PHQ-9)), physical illness, smoking status, physical activity, and education level QoL: quality of life; PE: premature ejaculation; ED: erectile dysfunction

QoL domain	Unadjusted mean difference (PE – ED)	95% CI	p-value	Adjusted mean difference (PE – ED)**	95% CI	p-value
Physical	+0.70	0.07 to 1.33	0.030*	+0.48	0.01 to 0.95	0.045*
Psychological	+0.75	0.06 to 1.44	0.032*	+0.52	0.02 to 1.02	0.041*
Social	+0.25	–0.31 to 0.82	0.392	+0.14	–0.35 to 0.63	0.578
Environmental	+0.06	–0.52 to 0.64	0.829	+0.08	–0.45 to 0.61	0.765

## Discussion

To the best of our knowledge, this is the first study on QoL among patients with PE and ED in Bangladesh. This single-center study included patients diagnosed with either PE (122) or ED (100), excluding those with both conditions. A small percentage of participants/patients were not diagnosed with PE and ED, which suggests that many individuals who do not meet the strict diagnostic criteria for PE but experience early ejaculation still seek medical help. Previously, Farahat et al. found 39% had mild ED and 8.28% had severe ED [15].

While South Asia is characterized by diverse cultural and religious practices, Bangladesh is predominantly influenced by Islamic beliefs, which shape societal norms around sexuality, modesty, and family life. Discussions of sexual health, particularly conditions such as PE and ED, are often stigmatized due to religious and moral conservatism [9]. This cultural backdrop may lead to underreporting of symptoms, reluctance to seek medical help, and psychological distress in men experiencing these issues. In this context, sexual dysfunction not only affects individual well-being but also carries significant implications for marital relationships and social identity, particularly where masculinity is closely tied to sexual performance [16]. In this study, participants from both groups reported the highest QoL in the physical domain and the lowest in the social domain. The physical domain often includes aspects such as general health, bodily functions, and vitality. In the context of PE and ED, physical health may be perceived as relatively intact compared to psychological and social well-being [17]. While these conditions are physically evident, their impact on daily physical activities is less severe than that of chronic diseases. However, the psychological and social effects tend to be more pervasive [18, 19]. The lowest QoL scores were observed in the social domain for both ED and PE patients, consistent with previous studies [19]. Sexual dysfunctions negatively impact interpersonal relationships, social interactions, and overall social functioning. Many individuals with ED and PE experience barriers to intimacy, leading to social withdrawal, relationship conflicts, and diminished self-confidence. This study also highlighted the presence of chronic conditions among PE and ED patients, emphasizing the complex relationship between chronic illnesses and sexual health. Hypertension was more prevalent among ED patients (21.0%) compared to PE patients (7.4%), likely due to its impact on vascular health and erectile function. Similarly, diabetes was observed in 14.0% of ED patients and 9.8% of PE patients, reflecting its role in nerve and blood vessel damage that contributes to sexual dysfunction [20, 21]. These findings underscore the importance of managing chronic illnesses to improve sexual health outcomes. 

A significant association was found between educational level and environmental well-being (p = 0.028), with higher education linked to better environmental well-being. This aligns with previous research suggesting that education enhances awareness and access to resources, leading to improved perceptions of environmental quality [22, 23]. Residence also influenced QoL, with urban dwellers reporting higher psychological (p = 0.030) and environmental well-being (p = 0.013) compared to those in suburban or rural areas. Cities provide greater access to healthcare, recreational facilities, and social opportunities, contributing to enhanced psychological and environmental well-being [24]. A significant difference in physical well-being was found between individuals with and without a history of physical illness (p = 0.032), with those without chronic conditions reporting higher scores. This supports prior research indicating that while chronic illnesses negatively affect physical health, individuals can maintain psychological and social well-being through effective coping strategies [25]. Lifestyle choices also played a role, with active individuals reporting significantly better environmental well-being (p = 0.028), consistent with studies linking physical activity to improved mood, reduced stress, and a greater sense of environmental control [26]. An unexpected finding was that smokers had significantly higher environmental well-being scores than non-smokers (p = 0.008). This contradicts existing literature, which typically associates smoking with poorer health outcomes [27]. One possible explanation is the social aspect of smoking, which may provide a sense of community and belonging [28]. However, further investigation is needed to explore the potential influence of social interactions, stress relief, and perceived control. Depression severity was negatively correlated with all QoL domains. Participants with severe depression reported significantly lower physical, psychological, social, and environmental well-being, with psychological well-being showing the steepest decline (p < 0.001). These findings align with existing literature demonstrating the detrimental impact of depression on cognition, emotional regulation, social engagement, and overall life satisfaction [29]. The findings indicate that men with PE have a better physical and psychological QoL than those with ED, even after controlling for confounders like depression severity (PHQ-9), physical illness, smoking status, physical activity, and education level, which is consistent with previous studies [30]. However, no significant differences were observed between the two groups in the social and environmental domains, highlighting that the impact of these sexual disorders may be more pronounced in specific areas of QoL rather than others.

Strengths of the study

Use of standardized and validated instruments enhances the reliability and validity of the findings. The analysis accounts for important covariates like depression severity, physical illness, smoking, physical activity, and education level, which strengthens the credibility of the conclusions.

Limitations of the study

This study has several limitations. First, its cross-sectional design precludes causal inferences, making it unclear whether sexual dysfunction leads to reduced QoL or vice versa. Second, as a single-center study, the findings may not be generalizable to broader populations due to potential differences in demographic or clinical characteristics. Selection bias may also be present, as individuals with both PE and ED were excluded, and those who chose to participate may differ systematically from those who declined. Additionally, reliance on self-reported measures for QoL and depression introduces the possibility of recall and social desirability bias.

## Conclusions

Depression emerged as the most significant negative factor, particularly affecting psychological and social QoL. In contrast, physical illness primarily impacted physical QoL, with minimal influence on social or psychological domains. An active lifestyle was positively associated with environmental QoL, whereas the effects of smoking remained inconclusive, potentially moderated by social influences. Participants with PE demonstrated significantly better physical and psychological QoL outcomes compared to those with ED. Our findings highlight the complex and multidimensional nature of QoL in PE and ED. In culturally conservative societies like Bangladesh, where sexual health discussions are often stigmatized due to religious and societal norms, the impact of these disorders may be even more profound and underreported. Further research is needed to explore the underlying mechanisms linking psychosocial and cultural factors with sexual dysfunction and quality of life. Longitudinal and multicenter studies could provide deeper insights into causal relationships and help develop culturally sensitive, tailored, holistic interventions.
